# Engineering of *Aspergillus niger* for the production of secondary metabolites

**DOI:** 10.1186/s40694-014-0004-9

**Published:** 2014-10-14

**Authors:** Lennart Richter, Franziska Wanka, Simon Boecker, Dirk Storm, Tutku Kurt, Özlem Vural, Roderich Süßmuth, Vera Meyer

**Affiliations:** 1 Institute of Chemistry, Department of Biological Chemistry, Berlin University of Technology, Straße des 17, Juni 124, Berlin, 10623 Germany; 2 Institute of Biotechnology, Department Applied and Molecular Microbiology, Berlin University of Technology, Gustav-Meyer-Allee 25, Berlin, 13355 Germany

**Keywords:** Aspergillus niger, Secondary metabolite, Nonribosomal peptide synthetase, Enniatin, Heterologous gene expression

## Abstract

**Background:**

Filamentous fungi can each produce dozens of secondary metabolites which are attractive as therapeutics, drugs, antimicrobials, flavour compounds and other high-value chemicals. Furthermore, they can be used as an expression system for eukaryotic proteins. Application of most fungal secondary metabolites is, however, so far hampered by the lack of suitable fermentation protocols for the producing strain and/or by low product titers. To overcome these limitations, we report here the engineering of the industrial fungus *Aspergillus niger* to produce high titers (up to 4,500 mg • l^−1^) of secondary metabolites belonging to the class of nonribosomal peptides.

**Results:**

For a proof-of-concept study, we heterologously expressed the 351 kDa nonribosomal peptide synthetase ESYN from *Fusarium oxysporum* in *A. niger*. ESYN catalyzes the formation of cyclic depsipeptides of the enniatin family, which exhibit antimicrobial, antiviral and anticancer activities. The encoding gene *esyn1* was put under control of a tunable bacterial-fungal hybrid promoter (Tet-on) which was switched on during early-exponential growth phase of *A. niger* cultures. The enniatins were isolated and purified by means of reverse phase chromatography and their identity and purity proven by tandem MS, NMR spectroscopy and X-ray crystallography. The initial yields of 1 mg • l^−1^ of enniatin were increased about 950 fold by optimizing feeding conditions and the morphology of *A. niger* in liquid shake flask cultures. Further yield optimization (about 4.5 fold) was accomplished by cultivating *A. niger* in 5 l fed batch fermentations. Finally, an autonomous *A. niger* expression host was established, which was independent from feeding with the enniatin precursor d-2-hydroxyvaleric acid d-Hiv. This was achieved by constitutively expressing a fungal d-Hiv dehydrogenase in the *esyn1*-expressing *A. niger* strain, which used the intracellular α-ketovaleric acid pool to generate d-Hiv.

**Conclusions:**

This is the first report demonstrating that *A. niger* is a potent and promising expression host for nonribosomal peptides with titers high enough to become industrially attractive. Application of the Tet-on system in *A. niger* allows precise control on the timing of product formation, thereby ensuring high yields and purity of the peptides produced.

**Electronic supplementary material:**

The online version of this article (doi:10.1186/s40694-014-0004-9) contains supplementary material, which is available to authorized users.

## Background

Recent genome mining efforts have uncovered that the genomes of filamentous fungi encode an unexpected rich repertoire of low-molecular-weight compounds with commercial relevance. These natural products known as secondary metabolites include nonribosomal peptides, polyketides and lipopeptides, which have pharmacological implications. Isoprenoids are interesting for the food industry as nutraceuticals or aroma compounds and poly-unsaturated fatty acids or lipids, can potentially be commercialized as biofuels. The natural product portfolio of filamentous fungi is thus impressive and emphasizes their great potential to become multi-purpose expression platforms in biotechnology. However, most of the genes involved in secondary metabolism pathways are not expressed under standard laboratory or industrial conditions and/or are present in intractable filamentous fungi, which prevents application of these natural products [1]-[3]. Different strategies based on molecular and epigenetics factors as well as cultivation methods have thus been undertaken to awaken these silent genes [4],[5]. In brief, secondary metabolite (SM) production is under control of complex regulatory gene networks and involves intricate multi-step biosynthetic machineries, as well as major reorganization of primary metabolic fluxes to redirect cellular metabolic resources towards their biosynthesis. SM expression is naturally linked with starvation-induced developmental processes leading to (a)sexual spore formation [6]–[8]. These processes can easily be tracked and even induced during bioreactor cultivations by adjusting low growth rates [9],[10].

The advent of synthetic biology opens new avenues to express any SM gene of interest in a filamentous fungal host which is easily tractable by genetic engineering. For example, the geodin gene cluster of *Aspergillus terreus* was recently reconstituted in *A. nidulans* and the penicillin cluster of *P. chrysogenum* was completely rewired and expressed from a polycistronic gene cluster under control of a single xylose-inducible promoter in *A. nidulans* [11]–[13]. Another system for *A. nidulans* is based on expression of any fungal SM gene of interest under control of an alcohol-inducible promoter and includes methods for deletion entire *A. nidulans* SM gene clusters. This approach is especially interesting as it eliminates production of the most abundant *A. nidulans* SMs, thus reducing the SM background and facilitating purification of the heterologously expressed SMs [14]. None of the inducible promoters used so far is tunable, carbon source-independent and tight under non-induced conditions. This, however, poses limitations in their use, especially when the switch in the carbon source affects changes in the primary metabolic fluxes which should provide precursors for heterologous SM production. This limitation, however, can be overcome by applying an artificial expression system based on the Tet-on system, which was established and systematically evaluated for use in *A. niger* but is functional in many other filamentous fungi [15]–[17]. The Tet-on system is a tunable bacterial-fungal hybrid expression system, which becomes induced upon addition of the tetracyclin-derivative doxycycline (Dox). Importantly, the inducing power depends on the Dox concentration applied and reaches expression levels which can compete with the strength of one of the strongest promoters known for filamentous fungi, the *gpdA* promoter from the glycolytic pathway [15].

We chose the industrial fungus *A. niger* as the expression host to determine whether or not the Tet-on system can be applied to produce high amounts of fungal SMs. Although the genome of *A. niger* carries the *pptA* gene encoding the key biosynthetic enzyme of fungal SM pathways (a 4′-phosphopantetheinyl transferase responsible for posttranslational activation of nonribosomal peptide synthetases and polyketide synthetase [18]), *A. niger* has so far only been exploited as expression platform for large-scale production of organic acids, proteins and enzymes [19]. Most importantly, the Tet-on system allows free choice over the timing of product formation as it can be switched on at any time during cultivation of *A. niger* [15]. We decided to induce heterologous SM expression during the exponential growth phase of *A. niger* due to two reasons. First, a maximum of ATP and primary metabolism intermediates are available during exponential growth phase, hence very high SM yields could supposedly be achievable. Second, endogenous SMs of *A. niger* become mainly expressed during carbon-starvation, i.e. during post-exponential growth phase [9],[10],[20]. Hence, heterologous SM production can be decoupled from homologous SM production and the *A. niger* cultures could largely be kept SM background-free.

**Figure 1:**
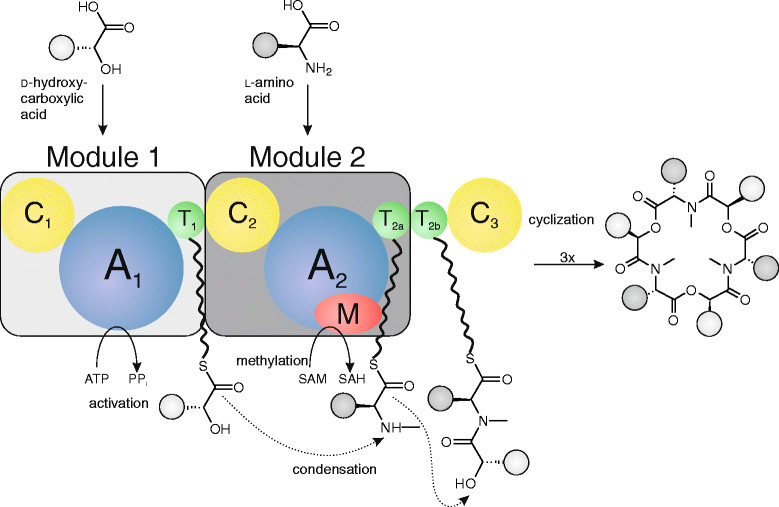
**Model of enniatin biosynthesis.** The precursors d-hydroxycarboxylic acids and l-amino acids become activated at the A_1_- and the A_2_-domain of the enniatin synthetase ESYN. *N*-methylation of the amino acid takes place at the M-domain. The building blocks are transferred from one module to another by means of T-domains and are ultimately stored at the “waiting position” T_2b_. Condensation of the building blocks and final cyclization and release from the enzyme is catalyzed by the C-domains. Modified after [21].

**Figure 2:**
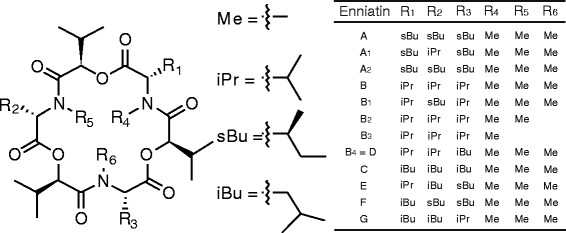
**Amino acid composition and methylation pattern of the enniatin family.** Enniatins are composed of three d-hydroxycarboxylic acids and three l-amino acids. The structural diversity is defined by the incorporation of different l-amino acids (R_1_-R_3_), which can be valine (iPr), leucine (iBu) or isoleucine (sBu). l-amino acids can be methylated (Me, R_4_-R_6_). Modified after [22].

For the proof-of-concept study, we decided to express the enniatin synthetase ESYN from *Fusarium oxysporum* in *A. niger*. Enniatin is a mixture of nonribosomal peptides and belong to the group of cyclic depsipeptides [21] which are mainly produced by the genus *Fusarium* (for reviews see [22],[23]). Enniatin is synthesized by the multifunctional enzyme ESYN, which uses three d-hydroxycarboxylic acids and three l-amino acids as precursors and requires the cofactors ATP and *S*-adenosylmethionine (Figure 1, [24],[25]). ESYNs from various *Fusarium* species use different amino acid precursors and display relaxed substrate specificities, which results in a wide spectrum of naturally occurring enniatins (Figure 2). After the first isolation of enniatin in 1947 [26], at least 29 naturally occurring derivatives were isolated from *Fusaria*.

Enniatin features antimicrobial [27], antiviral [28], cytotoxic [29] and phytotoxic [30] effects. For example, fusafungin, which is a mix of three derivatives (enniatin A, B and C), is a bactericide acting against gram-positive and gram-negative bacteria and is used as a topical agent for the treatment of respiratory infections [31]. The mode of action of enniatin is mainly linked to its ionophoric activity. It is known that enniatin B forms complexes with cations in the ratio 1:1, 2:1 or 2:3 and complexes K^+^, Ca^2+^, Na^+^, Mg^2+^ and Li^+^ [32], thereby forming cation-selective pores in biomembranes [33],[34]. Additionally, the bioactivity of enniatins can be linked to their inhibition of drug efflux pumps [35] and cholesterol acyltransferase activity [36].

The *F. oxysporum* enniatin synthetase ESYN synthesizes enniatin by coupling three d-hydroxycarboxylic acids and three l-amino acids via amide and ester bonds in an alternating fashion (Figure 2). Whereas ESYN can accept different amino acids as precursors (l-valine, l-isoleucine or l-leucine), only one species of d-hydroxycarboxylic acid can be found in natural enniatin isolates (d-hydroxyisovaleric acid, d-Hiv). d-Hiv stems from the l-valine metabolism: l-valine is deanimated by a valine aminotransferase to 2-keto-isovaleric acid (2-Kiv), which becomes subsequently reduced by a keto-isovaleric acid reductase (KivR) under consumption of NAD(P)H to d- Hiv. In contrast to *F. oxysporum*, *A. niger* has not been reported so far to produce enniatin. Although open reading frames with weak similarity to the *esyn1* gene of *F. oxysporum* are present in its genome (An01g11770, An08g02300, An11g00050, An12g07230, An13g03040, [20]), it lacks a *kivR* gene [20]. Hence, *A. niger* could potentially be able to produce enniatin, given that d-Hiv is present in the medium.

The main objective of this study was to determine whether *A. niger* is a suitable expression host for high-level production of fungal nonribosomal peptides. We therefore put the *esyn1* gene from *F. oxysporum* under control of the Dox-inducible Tet-on system and expressed it heterologously in *A. niger* to produce the enniatin as a model nonribosomal peptide. We optimized the production conditions using a design-of-experiment (DOE) approach which addressed medium composition, d-Hiv feeding conditions and Dox concentration. We furthermore optimized the cultivation conditions by establishing batch and fed batch cultures for an *esyn1* expressing *A. niger* strain. Finally, we engineered an autonomous enniatin B producing *A. niger* strain which is independent from d-Hiv feeding.

## Results

### Heterologous expression of the *esyn1* gene in *A. niger*

**Figure 3:**
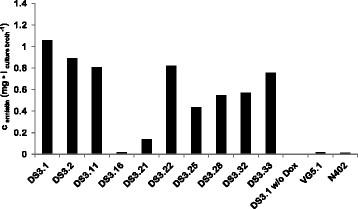
**Screening for the best enniatin producing strain.** 5 × 10^6^ spores/ml were cultivated in 20 ml complete medium for 40 h. Expression of the *esyn1* gene was induced after 16 h of cultivation time using 20 μg/ml Dox. From each strain, enniatin was purified from biomass and supernatant samples and the overall enniatin concentration harvested is indicated.

**Figure 4:**
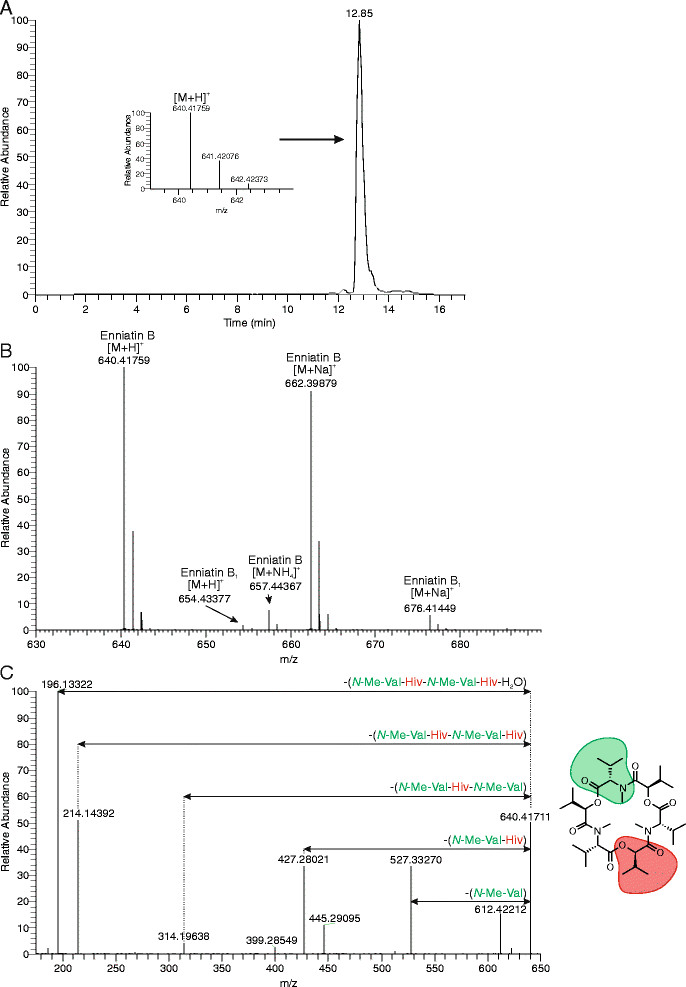
**Analysis of enniatins produced by strain DS3.1. A)** HR-LCMS chromatogram of purified enniatin B. A segment from the mass spectrum shows [M + H^+^] of enniatin B with the characteristic isotope pattern. The main peak can be assigned to enniatin B. Minor impurities can be detected at retention time 11.8 min and 12.2 min. **B)** HR-LCMS average mass spectrum. As example, the mass spectrum of purified enniatin B is shown. The H^+^, NH_4_^+^, Na^+^ adducts of enniatin B can be observed. The sample contains small amounts of enniatin B1. Samples were measured on an ESI-Orbitrap-MS. **C)** ESI-HRMS/MS spectrum obtained with a LTQ Orbitrap XL apparatus using direct injection and applying a collision energy of 12 eV. The moiety highlighted in green represents the l-valine and the moiety highlighted in red represents d-Hiv incorporated into the enniatin B structure. For the fragments *m/z* values were calculated. The calculated *m/z* value for the [C_27_H_47_N_2_O_8_]^+^ fragment is 527.33269 and the *m/z* value observed was 527.33270. For the [C_22_H_39_N_2_O_6_]^+^ fragment, the calculated *m/z* value was 427.28026 and the *m/z* value observed was 427.28021. The calculated *m/z* value for the [C_16_H_28_NO_5_]^+^ fragment was 314.19620 and the *m/z* value measured was 314.19638. The calculated *m/z* value for the [C_11_H_20_NO_3_]^+^ fragment was 214.14377 and the *m/z* value observed was 214.14392.

The *esyn1* gene of *F. oxysporum* was integrated in plasmid pVG2.2 [15] to give plasmid pDS4.2, which comprises all three components of the Tet-on system: *PgpdA::rtTA2*^*S*^ *-M2* for constitutive expression of the transactivator rtTA, *tetO7::Pmin::esyn1*, which mediates *esyn1* expression in a Dox-dependent manner (note that rtTA is only able to bind to its operator sequence *tetO7* when bound to Dox, [15]) and the pyrG* cassette, necessary for selection and targeting of the system to the *pyrG* locus of *A. niger* (Additional file 1: Figure S1). As recipient strain, the protease-negative (*prtT*^−^) and uracil-auxotroph (*pyrG*^*−*^) strain AB1.13 [37] was used. Uridine-prototroph transformants were selected and screened via PCR and Southern analysis for the presence of single or multiple pDS4.2 copies in the genome of *A. niger* (Additional file 1: Figure S1 and data not shown). Ten pDS4.2-carrying transformants were selected and cultivated in liquid shake flask cultures in the presence or absence of Dox. Controls were an *A. niger* wild type strain (strain N402), the original producer *F. oxysporum* (strain ETH1536) and an *A. niger* strain harboring a single copy of the *esyn1*-free plasmid pVG2.2 at the *pyrG* locus (strain VG5.1). After cultivation, enniatin was isolated from the biomass and the culture supernatant by means of ethyl acetate extraction. The amount of enniatin produced was determined and quantified by HPLC-MS. Among the transformants, strain DS3.1, which carried a single copy of pDS4.2 at the *pyrG* locus, produced the highest amount of enniatin (about 1 mg • l^−1^, Figure 3). Only minute amounts of enniatin were detectable in the control strains N402 and VG5.1 and all pDS4.2 carrying strains in the absence of Dox, verifying that the expression system is tight under non-induced conditions. The *m/z* values and retention time of enniatin isolated from the different *A. niger* transformants were equal to those extracted from the natural enniatin producer *F. oxysporum* (Figure 4 and data not shown). Several derivatives could be detected and characterized by tandem MS, amongst them enniatin A, A_1_, B and B_1_ (data not shown). However, because the standard used was a mixture of enniatin A, B, and C isolated from *F. oxysporum*, the exact ratio and amount of the enniatin variants could not be determined. However, full MS-scans of the standard and DS3.1 samples showed that the enniatin composition was similar to *F. oxysporum* (Figure 4).

### Optimization of enniatin production

**Figure 5:**
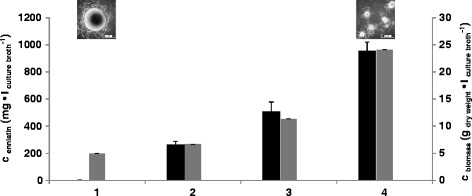
**Optimization of the enniatin yield.** 5 × 10^6^ spores/ml of strain DS3.1 were cultivated in 20 ml shake flask cultures containing complete medium with varying composition. Induction of *esyn1* expression was performed in all media with 10 μg/ml Dox. Selected results are exemplarily shown: (1) 0 mM l-valine/l-isoleucine/l-leucine, 1% glucose, 0 g/l talcum, 40 h cultivation time, 30°C cultivation temperature. (2) 10 mM l-valine/l-isoleucine/l-leucine, 1% glucose, 0 g/l talcum, 10 mM d-Hiv, 92 h cultivation time, 26°C cultivation temperature. (3) 10 mM l-valine/l-isoleucine/l-leucine, 2.5% glucose, 2.5 g/l talcum, 10 mM d-Hiv, 92 h cultivation time, 26°C cultivation temperature. (4) 20 mM l-valine/l-isoleucine/l-leucine, 5% glucose, 10 g/l talcum, 10 mM d-Hiv, 92 h cultivation time, 26°C cultivation temperature. The total enniatin concentration (black bars) and biomass concentration (grey bars) is given. Data from biological triplicates are shown. Microscopic pictures of DS3.1 pellets are shown. Bar, 500 μm.

In order to identify the optimum condition for high yield production of enniatin, a design-of-experiment approach was followed using the statistical software program MODDE (see Methods). The following parameters were varied in 20 ml shake flask cultures of the *esyn1*-expressing strain DS3.1: (i) medium composition (minimal medium, complete medium, *Fusarium* defined medium) [38], (ii) l-valine, l-leucine, l-isoleucine supplementation (0, 10, 20 mM), (iii) d-Hiv supplementation (0, 5, 10, 50 mM), (iv) glucose concentration (1, 2.5, 5%), (v) temperature (26°C, 30°C), (vi) cultivation time (24, 36, 48, 92 h) and (vii) Dox concentration (0, 5, 10, 20 μg/ml). The parameters which mainly affected enniatin yields were Dox and d-Hiv (data not shown) and the best cultivation medium identified contained 20 mM d-Hiv, 20 mM of one of the amino acids and 10 μg/ml Dox. This medium composition improved the enniatin yield by a factor of 200 to 200 mg ∙ l^−1^ (Figure 5), whereby most of the enniatin could be extracted from biomass samples after 92 h of cultivation. Remarkably, the enniatin yield was further increased about 4.75-fold by increasing the glucose concentration to 5% and by adding talcum to the DS3.1 cultures (Figure 5). As reported recently, the addition of microparticles to liquid cultures of *A. niger* reduces the diameter of macromorphological pellets to only a few hundred micrometers. This in turn considerably improves uptake rates of nutrients and oxygen and increases the metabolic activity of *A. niger* [39]. Taken together, the final enniatin yield was 950 mg · l^−1^ culture broth (corresponding to 0.04 g · g^−1^ dry weight biomass).

### Modulation of the enniatin product variety by targeted supplementation with amino acids

**Figure 6:**
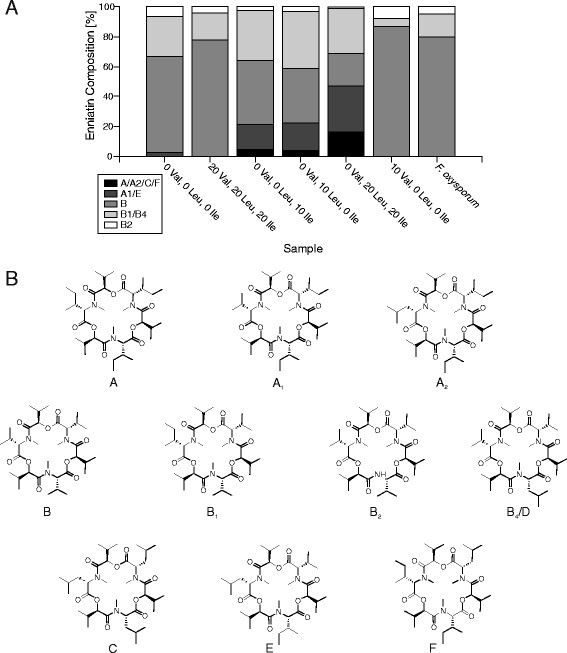
**Spectrum of enniatin species produced by strain DS3.1. A)** Different enniatin variants were produced depending on the amino acids fed. The final concentration of l-valine, l-leucine l-isoleucine in the cultivation medium is given in mM. **B)** Structures of enniatin species produced in strain DS3.1.

In order to determine the scope of enniatin variants, which can be heterologously synthesized by *A. niger*, the precursor amino acids l-valine, l-leucine and l-isoleucine were added to DS3.1 cultures either individually or in combination. After cultivation for 72 h, the profile of the synthesized enniatins was determined by HPLC-MS. When no or all three amino acids were added at the same concentration, the main products were enniatin B, B1 and B4, whereas enniatins A, A1, A2, C, E and F were only present in trace amounts (Figure 6A). The latter fraction increased, when l-leucine or l-isoleucine were added and reached almost 50% of total enniatin, when both amino acids were supplemented each at 20 mM. When the cultivation medium was supplemented with l-valine only, the main product was enniatin B (87%). Interestingly, enniatin variants containing d-lactate moieties were also synthesized by strain DS3.1, which have so far not been observed in *F. oxysporum*. This suggests that the precursor repertoire which can be used by ESYN is broader in *A. niger* compared to *F. oxysporum*.

### Isolation and analytics of enniatin B

The identity and purity of enniatin produced by strain DS3.1 was confirmed by ^1^H-NMR-, ^13^C-NMR-, IR-, MS-, MS/MS- and X-ray analysis (Additional file 2: Figure S2 and data not shown). From a 1 liter shake flask culture (20 mM d-Hiv, 20 mM l-valine, 5% glucose, 10 μg/ml Dox, 10 g/l talcum), 800 mg enniatin could be isolated by crystallization, which corresponded to an enniatin yield of 0.04 g · g^−1^ dry weight biomass. The majority of enniatin was isolated from the biomass. The crystallized enniatin had a dark yellowish to brownish color and enniatin variats could be separated e.g. by means of preparative HPLC. After repetitive multiple crystallizations, a total of 675 mg enniatin were obtained. The NMR spectra were identical to the ones published in literature [32],[40] and proved that the crystals were pure enniatin B as summarized as follows: ^1^H-NMR spectrum: (400.1 MHz, CDCl3) *δ* = 5.11 (d, ^3^ *J*_H,H_ = 8.7 Hz, 3 H), 4.49 (d, ^3^ *J*_H,H_ = 9.7 Hz, 3 H), 3.11 (s, 9 H), 2.32-2.21 (m, 6 H), 1.05 - 0.86 ppm (m, 36 H). ^13^C-NMR spectrum: (100.6 MHz, CDCl_3_) *δ* = 170.25, 169.31, 75.67, 63.20, 33.26, 29.91, 27.92, 20.42, 19.34, 18.72, 18.50 ppm. IR spectrum (Neat): *υ* = 2963.6-2873.4 (C-H, CH_3_ and CH), 1736.1 (C = O, ester), 1660.9 (C = O, amide), 1183.6 (C-H, isopropyl) 1011.0 (CO, *α*-hydroxycarboxylic acid). ESI-HRMS spectrum: *m/z* calculated for [C_33_H_57_N_3O9_ + Na]^+^: 662.39870; found: 662.39859; ESI-HRMS/MS: *m/z* calculated for [C_27_H_47_N_2_O_8_]^+^: 527.33269; found: 527.33221, *m/z* calculated for [C_22_H_39_N_2_O_6_]^+^: 427.28026; found: 427.27988, *m/z* calculated for [C_16_H_28_NO_5_]^+^: 314.19620; found: 314.19614, *m/z* calculated for [C_11_H_20_NO_3_]^+^: 214.14377; found: 214.14375. The masses of the daughter ions are due to cleavages at the ester and amide bonds (*m/z* = 527.33, 427.28, 314.20, 214.14) as described by [41].

The X-ray crystallographs demonstrated that the crystals had no impurities and were a complex of enniatin B with Na^+^ ions, whereby one Na^+^ ion was located in the center of an enniatin B molecule (Additional file 2: Figure S2). As a result, the adjoining molecule from the next layer in the crystal is not located on the same axis but is shifted to the side. Thus, sandwich structures of enniatin B with the Na^+^ ions were not formed.

### Production of enniatin B by batch and fed batch bioreactor cultivation

**Figure 7:**
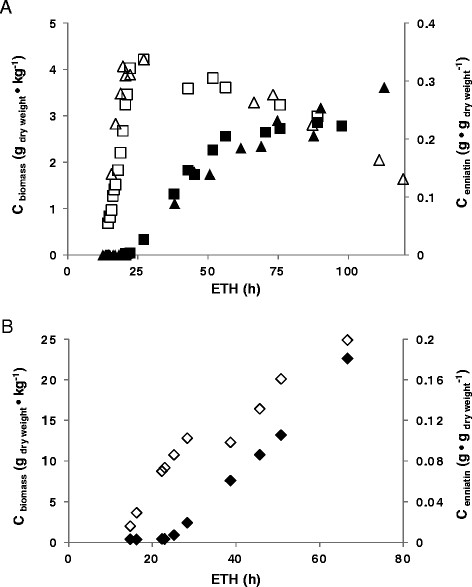
**Submerged batch and fed batch cultivation of strain DS3.1. A)** Biomass (open symbols) and enniatin accumulation (closed symbol) of two batch cultivations are shown. **B)** Biomass (open symbol) and enniatin accumulation (closed symbol) of a fed batch cultivation are shown.

In order to obtain high enniatin yields under controlled conditions in bioreactors, 5 l batch cultivations of strain DS3.1 were performed using a defined fermentation medium. This medium had a pH of 3 and was balanced as such, that glucose was the growth-limiting nutrient (final concentration 0.8%; see Methods). Note that the low pH of the medium and the use of ammonia as nitrogen source ensures dispersed morphology of *A. niger* during bioreactor cultivation with no need for adding microparticles [42]. After the culture reached the early exponential growth phase (corresponding to 1 g biomass dry weight · l^−1^ culture broth after about 14–16 h post inoculation), production of enniatin was induced by the addition of 10 μg/ml Dox, 20 mM d-Hiv and 20 mM l-valine, respectively. In two independent runs, the maximal specific growth rate achieved was 0.24 h^−1^ and the biomass concentration peaked at 4.2 g · kg^−1^ culture broth after about 26 h post inoculation (Figure 7A). During exponential growth, pH 3 was maintained by the addition of 1 M NaOH, which has been shown to linearly correlate with the biomass accumulation and reflecting ammonium uptake during balanced growth [9],[43]. The end of the exponential growth phase was detected by an increase of the dissolved oxygen signal (data not shown), after which the cell mass decreased by nearly 50% (Figure 7A). Importantly, the levels of CO_2_ and O_2_ in the exhaust gas clearly indicated that the cultures were still metabolically active, even 100 hours after depletion of the carbon source (data not shown). As recently demonstrated, carbon starvation of *A. niger* during submerged cultivation results in secondary growth by carbon recycling leading to a gradual transition from old to young hyphae [9]. Enniatin levels determined for selected time points demonstrated that enniatin B was mainly produced after carbon source depletion (i.e. after about 55 h post inoculation) and reached a maximum value of 0.29 g · g^−1^ dry weight biomass after about 110 h of cultivation (Figure 7A).

Finally, one fed batch cultivation was performed to increase biomass concentration and thereby enniatin B yield. After the culture reached the late exponential growth phase (corresponding to 4 g biomass · kg^−1^ culture broth after about 18 h post inoculation), expression of the *esyn1* gene was induced by feeding with 5% glucose, 10 μg/ml Dox, 20 mM d-Hiv and 20 mM l-valine. To ensure that the *esyn1* gene was continuously expressed at highest level, 10 μg/ml Dox were added every 4–7 h resulting in a final Dox concentration of 90 μg/ml. As depicted in Figure 7B, this fermentation protocol ensured a specific growth rate of 0.15 h^−1^ and the biomass concentration reached 24.9 g · kg^−1^ culture broth after about 66 h post inoculation. Enniatin B production started immediately after Dox induction and reached a maximum of 4.5 g · kg^−1^ culture broth after 66 h of cultivation (corresponding to 0.18 g · g^−1^ dry weight biomass, Figure 7B). Interestingly, linear accumulation of biomass in the fed batch run was paralleled by linear accumulation of enniatin B (Figure 7B), suggesting that the dynamics of enniatin B production under the Tet-on system displays primary metabolite kinetics.

### Establishment of an autonomous *A. niger* production strain

As mentioned above, *A. niger* cannot produce enniatin autonomously as its genome lacks the *kivR* gene encoding the d-Hiv generating enzyme α-ketoisovalerate reductase. In order to engineer an autonomous enniatin production strain which is independent from d-Hiv feeding, strain DS3.1 was transformed with the *kivR* gene from *F. oxysporum* (E-KivR), which was put under control of the constitutive *gpdA* promoter (see Methods). Three transformants carrying multi-copy integrations of the *PgpdA::kivR* construct (strains ÖV4.3, ÖV4.10, ÖV4.11, data not shown) were analyzed for enniatin production using 20 ml shake flask cultures. After induction with a final concentration of 10 μg/ml Dox and supplementation of 20 mM l-valine, the enniatin yield in all three transformants reached 0.03 g · g^−1^ dry weight biomass after 92 h of cultivation (data not shown), which is about 75% of the enniatin yield of strain DS3.1 when fed with 20 mM d-Hiv and 20 mM l-valine.

## Discussion

The fungal kingdom of approximately 1.5 million species exhibits a huge reservoir of secondary metabolites that span a broad variety of structurally and chemically diverse natural products. This reservoir has and will increasingly have a considerable potential impact on human welfare. The need to identify and produce novel bioactive fungal products goes far beyond antimicrobials and includes the requirement for novel drugs for various human health problems ranging from different cancers to neurodegenerative diseases, which are particularly emerging in aging societies. The newly emerged fungal ‘omics’ era and the advent of systems and synthetic biology provide innovative concepts and ideas to harness this untapped potential and to produce novel bioactive compounds from fungi on an industrial scale. The gene(s) or gene cluster(s) of interest can be of fungal or even (non)fungal origin and respective expression constructs can be plugged into fungal chassis strains allowing high expression levels. The gene(s) of interest will be expressed under the control of synthetic promoters and the product repertoire, the timing of product formation and productivity can be optimized using metabolic engineering strategies.

The aim of this study was to explore the potential of *A. niger* to become an expression system for secondary metabolites from other organisms. *A. niger* is so far being used in biotechnology as cell factory for the production of organic acids and secreted proteins [1]. For the proof-of-concept study, the cyclic depsipeptide enniatin was chosen. Heterologous production of cyclic depsipeptides such as beauvericin has been achieved in microbial host strains but only with very low yields, ranging from 3 mg · l^−1^ in *Escherichia coli* to 100 mg · l^−1^ in *Saccharomyces cerevisiae* [44],[45]. Heterologous expression of the *F. oxysporum esyn1* gene in *E. coli* was accomplished as well; however, only minute amounts were produced (1 mg · l^−1^; own unpublished data). Although chemical synthesis of enniatin is in principle possible, it can by far not fulfill the requirements for an efficient large-scale drug production process since its synthesis is too complex and very cost-intensive [46]. The pharmaceutical relevance of enniatin stimulated studies to use homologous fungal hosts such as *F. sambucinum* for enniatin production ([47],[48]). Although high amounts of enniatin were produced (1.7 g · l^−1^), these surface cultures lasted up to 5 weeks. Submerged shake flask cultivation of a randomly mutagenized *F. oxysporum* strain (strain ETH 1536) which included additional amino acid feeding resulted in the highest enniatin titer reported so far (5 g · l^−1^ after 96 h of cultivation, [38]).

Here, we demonstrate that heterologous expression of the *esyn1* gene under control of the Tet-on system in *A. niger* allows enniatin production rates which are considerably higher than ever reported for a heterologous host. 4.5 g · l^−1^ have been reached after 66 h of a fed batch cultivation of strain DS3.1, a yield which is sufficient for rapid scale-up, biological testing and commercial production. The yields which can be achieved with *A. niger* nearly reach the titer of the original production strain *F. oxysporum*. Two explanations might explain why *A. niger* is well suited for heterologous enniatin (and other nonribosomal peptides) production. First, *A. niger* possesses an endogenous PPTase, which is key for the posttranslational activation of NRPS [18]. Second, the ability to synthesize secondary metabolites is conserved in filamentous fungi. Their secondary metabolites are adaptive traits that have been subjected to natural selection during evolution. Although their occurrence apparently reflects particular life style and survival strategies and differ among fungal species, multiple secondary metabolic pathways are prevalent in each filamentous fungus. The existence of these pathways predestines filamentous fungi as hosts for heterologous fungal secondary metabolite production.

The genome of *A. niger* harbors five open reading frames (An01g11770, An08g02300, An11g00050, An12g07230, An13g03040), which display a weak similarity to the *esyn1* gene of *F. oxysporum* [20]. Analysis of their expression profiles using published transcriptomics data from *A. niger* cultures subjected to carbon-limited growth in batch, chemostat and retentostat bioreactor fermentations [9],[49],[50] revealed that An01g11770, An11g00050, An12g07230 and An13g03040 are silent under these conditions. An08g02300 though is expressed at low levels, with mean expression values of 10% or less when compared with the actin gene (An15g00560; data not shown). Hence, *A. niger* is likely to have the metabolic pathways and flexibility to synthesize enniatin with an *esyn1* gene being either of endogenous or exogenous origin. However, as it does not comprise the *kivR* gene encoding a d-Hiv dehydrogenase, feeding with d-Hiv or heterologous expression of a *kivR* gene is key to obtain high enniatin levels with *A. niger* as shown in this study. It has to be mentioned that An11g09950 shares high similarity with the d-Hiv dehydrogenase from *Gibberella intermedi.* An11g09950 is a predicted 2-dehydropantoate 2-reductase catalyzing a similar reaction as KivR, which is the reduction of 2-dehydropantoate to the d-hydroxycarboxylic acid d-pantoate under consumption of NADPH [50]. Due to the relatively high degree of similarity of both enzymes and their substrates, it might be conceivable that An11g09950 could accept α-ketoisovalerate as substrate to synthesize d-Hiv. This would explain why enniatin is present in minute amounts in strain DS3.1 cultures when not fed with d-Hiv. In any case, establishment of an autonomous *A. niger* strain being independent of d-Hiv feeding will considerably reduce the cost of the fermentation process. Our data clearly demonstrated that heterologous expression of the *kivR* gene from *F. oxysporum* rendered *A. niger* autonomous with respect to d-Hiv feeding and allowed high level enniatin production.

Different cultivation protocols were run in this study to heterologously produce enniatin in strain DS3.1. The specific yields of enniatin accomplished in shake flask cultures were 0.04 g · g^−1^ dry weight biomass (20 ml and 1 l cultures) and 0.18 g · g^−1^ dry weight biomass during the fed batch cultivation. In these cultivations, 5% glucose served as carbon source. The dynamics of enniatin B production clearly displayed primary metabolite kinetics in the fed batch run (Figure 7B), proving that the Tet-on system can ensure high level *esyn1* expression during exponential growth. The highest specific enniatin yield, however, was observed for the batch cultivation (0.29 g · g^−1^ dry weight biomass), which used only 0.8% glucose as carbon source. Interestingly, the enniatin production followed secondary metabolite kinetics although the Tet-on system was switched on early during exponential growth phase (Figure 7A). Carbon-induced starvation has clearly been demonstrated to induce a plethora of secondary metabolites in *A. niger* [9],[50]. It is thus conceivable that the transition to the post-exponential growth phase might have caused a metabolic shift in *A. niger* which favored secondary metabolite production in general and enniatin production in particular. The overall activated secondary metabolite machinery of *A. niger* might have strongly supported enniatin expression, e.g. by providing additional carbon and nitrogen due to autophagy [9], by ensuring a higher amino acid pool and/or by increasing the stability of the *esyn1* transcript or the ESYN protein. Future studies are necessary to understand these processes; clearly, only systems-level insights will help to elucidate the molecular mechanisms behind.

## Conclusions

This is the first report demonstrating that *A. niger* is a potent expression host for nonribosomal peptide synthetase. The strong inducibility of the Tet-on system combined with controlled bioreactor cultivation allowed the production of enniatin with yields which are high enough to become industrially relevant.

## Methods

### Strains, media and molecular techniques

*Aspergillus* strains used in this study are given in Table 1. Strains were grown on minimal medium (MM) [51] containing 1% (w • v^-1^) glucose and 0.1% (w • v^−1^) casamino acids or on complete medium (CM), containing 0.5% (w • v^−1^) yeast extract in addition to MM. When required, plates were supplemented with uridine (10 mM). Transformation of *A. niger* and fungal chromosomal DNA isolation was performed as described [52]. All molecular techniques were carried out as described earlier [53].

**Table 1:** **Strains used in this study**

Strain	Relevant genotype	Source
*Fusarium oxysporum*		
ETH1536	Wild type	[54]
*Aspergillus niger*		
N402	Wild type	[55]
AB1.13	*pyrG* ^*−*^ *, prtT* ^*−*^	[37]
VG5.1	*pyrG*^*+*^, ∆*kusA* (transformed with pVG2.2; single copy)	[15]
DS3.1	*pyrG*^*+*^ *, prtT*^*−*^ *, esyn1* (transformed with pDS4.2, single copy)	This work
ÖV4.3	*pyrG*^+^, *prtT*^−^, *esyn1, EkivR* (transformed with pÖV2.3; multi copy)	This work
ÖV4.10	*pyrG*^+^, *pyrtT*^−^, *esyn1, EkivR* (transformed with pÖV2.3; multi copy)	This work
ÖV4.11	*pyrG*^+^, *prytT*^−^, *esyn1, EkivR* (transformed with pÖV2.3; multi copy)	This work

The coding sequence of ESYN was PCR-amplified from a fosmid library of *F. oxysporum* ETH 1536 and ligated into the PmeI-linearized plasmid pVG2.2 (P*gpdA*::rtTA::T*cgrA*-*tetO7*::Pmin::T*trpC*-pyrG*, [15]). The resulting plasmid was named pDS4.2. The *kivR* gene from *F. oxysporum* ETH 1536 was PCR-amplified and ligated into the expression vector pNOM102 [56] via NcoI restriction. Thereby, the ß-glucuronidase gene was replaced by *kivR*. The resulting plasmid was named pÖV4.1. The protease-deficient *A. niger* strain AB1.13 was transformed with pDS4.2 using its uracil-auxotrophy for selection [57]. Strain DS3.1 was co-transformed with plasmid pÖV4.1 and the selection plasmid p3SR2, which expresses acetamidase (*amdS*) as selection marker. Transformation of *A. niger* and fungal chromosomal DNA isolation was performed as described [52]. All molecular techniques were carried out as described earlier [53].

### Optimization of enniatin production

Optimum cultivation conditions for enniatin production were identified using the statistical software program MODDE 9.1 (Umetrics). The screening experiments were performed in 20 ml of CM which were inoculated with 5 × 10^6^ spores · ml^−1^ of strain DS3.1. All cultivations were performed at 250 rpm. After 16 h (~1 g dry weight · l^−1^), enniatin expression was induced by the addition of different concentrations of Dox. Cultures were harvested after 24 h by filtration and defined amounts of biomass and supernatant were used to isolate enniatin. Several cycles of optimization were performed which included the parameters cultivation time, concentration of inductor and d-Hiv, type of the cultivation medium, temperature, concentration of l-Val, l-Leu, and l-Ile, concentration of carbon source and talcum concentration (−350 mesh). As optimum cultivation condition was eventually identified: CM containing 5% glucose and 10 g/l talcum, temperature 26°C, addition of 20 mM d-Hiv, 20 mM l-Val and 10 μg/ml Dox after 16 h of cultivation, total cultivation time 92 h.

### Extraction and purification of enniatin

Defined amounts of DS3.1 supernatant and biomass cultures were extracted with ethyl acetate. Extracts were centrifuged, dried over Na_2_SO_4_ and the solvent evaporated. Samples were dissolved in methanol, diluted if necessary and the enniatin concentration determined by HPLC-MS analysis. The HPLC-MS measurements for quantification were performed on an ESI-Triple-Quadrupol-MS, 6460 Series (Agilent Technologies) in multiple reaction monitoring mode. The utilized column was an Eclipse Plus C18, 2.1×50 mm column (Agilent Technologies) and the mobile phases were H_2_O + 0.1% formic acid (A) and acetonitrile + 0.1% formic acid (B). The injection volume was set to 2 μl and the flow rate was 0.3 ml/min. The *m/z* value for the precursor ion was set to 640.4 (*m/z* of [enniatin B H^+^] - adduct) and for the fragment ions to 527.4 as quantifier, 427.3 and 196.2 as qualifier. For every set of measurements, a new calibration curve was made using enniatin isolated from *F. oxysporum* as an external standard. Peak areas were determined by manual integration using masshunter workstation quantitative analysis (Agilent).

For purifying enniatin by recrystallization, the crystals were resolved in a minimal amount of hot ethyl acetate. Acetonitrile was slowly added until clear crystals started to appear. The mother liquor was decanted and the crystals were washed several times with acetonitrile. Enniatin obtained from crystallization was applied to preparative HPLC (1100 series, Agilent Technologies) running isocratically 70% methanol, containing 0.1% formic acid on a C18-column (Grom-Sil 120 ODS-5 ST, 10 μm, 250 × 20 mm, Grace).

### Identification and characterization of enniatin B

^1^H-NMR and ^13^C-NMR spectra of enniatin B were recorded on a Bruker Avance 400 NMR-spectrometer. The signals of the non-deuterated solvent rests were used as standards. Chemical shifts are given in δ-units (ppm) relative to the solvent signal. IR spectra were recorded on a Jasco FT-IR 4100 spectrometer. High-resolution mass-spectrometry (HRMS) using ESI-technique was performed on a LTQ Orbitrap XL apparatus. Data for the single-crystal structure determination of enniatin B were collected on an Oxford-Diffraction Xcalibur diffractometer, equipped with a CCD area detector Sapphire S and a graphite monochromator utilizing MoK_α_ radiation (λ = 0.71073 Å). Suitable crystals were attached to glass fibers using per-fluoropolyalkylether oil and transferred to a goniostat where they were cooled to 150 K for data collection. The software packages used were CrysAlis CCD for data collection, CrysAlis Pro for cell refinement and data reduction.

### Bioreactor cultivation

Submerged cultivations were performed with 6.6 liter BioFlo3000 bioreactors (New Brunswick Scientific, NJ, USA) and a detailed description of the fermentation settings was previously given 10. In brief, glucose-limited batch cultivation was initiated by inoculation of 5 l (kg) fermentation medium with conidial suspension of strain DS3.1 to give 10^9^ conidia l^−1^. Glucose was sterilized separately from the fermentation medium and final concentration was 0.8% (w • v^−1^). Temperature of 30°C and pH 3 were kept constant, the latter by computer controlled addition of 2 M NaOH or 1 M HCl, respectively. Acidification of the culture broth was used as an indirect growth measurement [58]. When the culture reached the early exponential growth phase (corresponding to 1 g biomass dry weight · kg^−1^), Dox (10 μg/ml), d-Hiv (20 mM) and l- Val (20 mM) were added.

The fed batch cultivation was started with 4 l fermentation medium. Induction of the Tet-on system with Dox and addition of feeding medium (FM, 0.046 l • h^−1^) was started when the culture reached the late exponential growth phase. FM is composed of fermentation medium with 5% glucose, 0.5% YE, 0.1% casamino acids, 20 mM d-Hiv and 20 mM l-valine. Every 4–7 h, 10 μg/ml of Dox were added.

## Availability of supporting data

The data sets supporting the results of this article are included within the article and its additional files.

## Additional files

## Electronic supplementary material

Additional file 1: Figure S1.: Southern analysis of *A. niger* transformants. (TIFF 667 KB)

Additional file 2: Figure S2.: COSY NMR-spectrum and crrystal structure of enniatin B. (JPEG 485 KB)

Below are the links to the authors’ original submitted files for images.

Authors’ original file for figure 1

Authors’ original file for figure 2

Authors’ original file for figure 3

Authors’ original file for figure 4

Authors’ original file for figure 5

Authors’ original file for figure 6

Authors’ original file for figure 7
