# Supplementary material for: Engineering of Aspergillus niger for the production of secondary metabolites
Source: Fungal Biol Biotechnol. 2014 Oct 14;1:4. doi: 10.1186/s40694-014-0004-9 (PMC5598268; doi:10.1186/s40694-014-0004-9)
Supplement: Supplementary file 1 — Additional file 1: Figure S1.: Southern analysis of A. niger transformants. (TIFF 667 KB) [file 40694_2014_4_MOESM1_ESM.tiff]

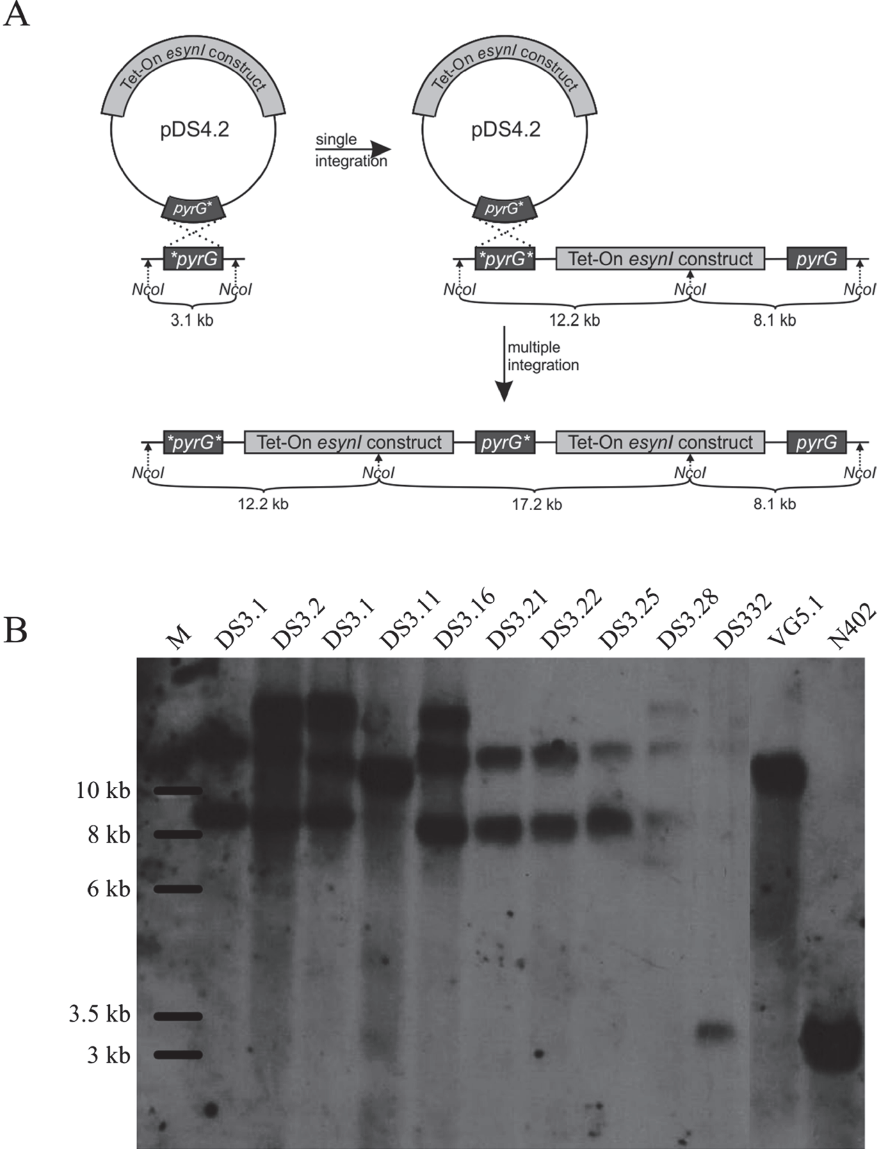

To confirm homologous integration of the constructs at the *A. niger pyrG* locus, genomic DNAs of selected transformants were restricted with NcoI and subjected to Southern hybridization using *pyrG* as a probe. Strain N402 served as a wild type control. (A, B) The expected fragment size for the wild type *pyrG* is 3.1 kb. *For* a single-copy integration of construct pDS4.2 at *pyrG*, two signals are expected (12.2 kb, 8.1 Kb). For a tandem-copy integration of construct pDS4.2 at *pyrG*, three signals are expected (12.2 kb, 8.1 Kb, 17.2 kb).
