# Supplementary material for: Engineering of Aspergillus niger for the production of secondary metabolites
Source: Fungal Biol Biotechnol. 2014 Oct 14;1:4. doi: 10.1186/s40694-014-0004-9 (PMC5598268; doi:10.1186/s40694-014-0004-9)
Supplement: Supplementary file 2 — Additional file 2: Figure S2.: COSY NMR-spectrum and crrystal structure of enniatin B. (JPEG 485 KB) [file 40694_2014_4_MOESM2_ESM.jpeg]

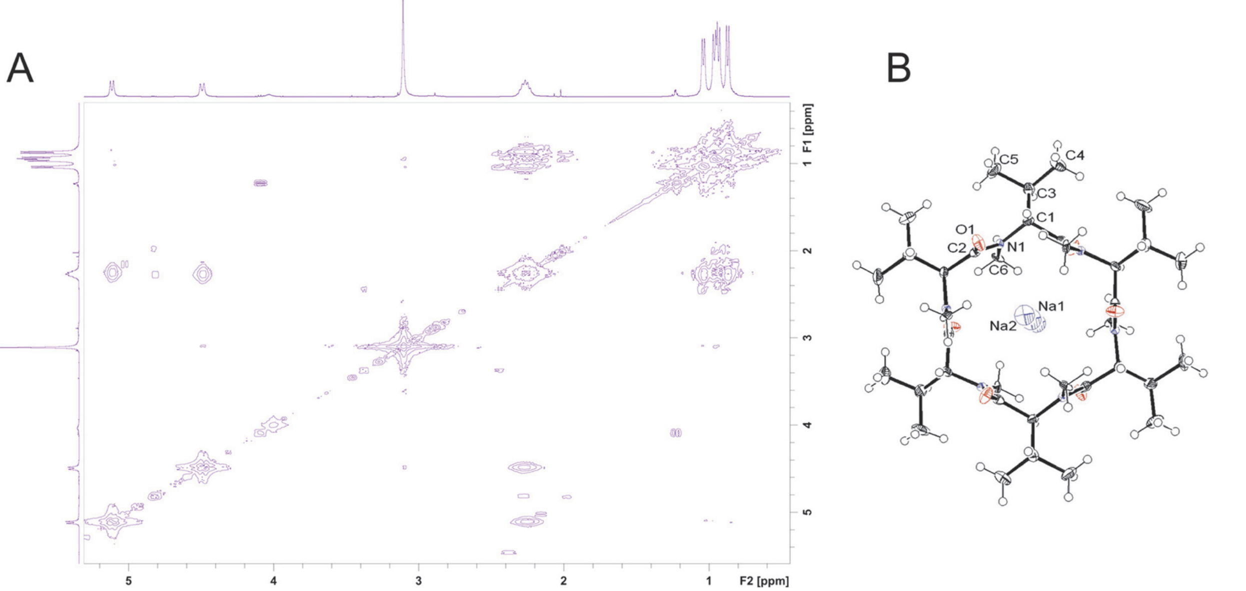

A. The COSY spectrum of enniatin B was recorded on a Bruker Avance 400 NMR-spectrometer.
B. Data for the single-crystal structure determination of enniatin B were collected on an Oxford-Difraction Xcalibur difractometer, equipped with a CCD area detector Sapphire S and a graphite monochromator utilizing MoKα radiation (λ = 0.71073 Å). Suitable crystals were attached to glass fibers using per-fluoropolyalkylether oil and transferred to a goniostat. The sample was cooled to 150 K for data collection. Software packages used: CrysAlis CCD fordata collection, CrysAlis Pro for cell refinement and data reduction. The conformation of a single enniatin B molecule is shown. Enniatin B cocrystalized with sodium.
