# Supplementary figures and images for: Engineering of Aspergillus niger for the production of secondary metabolites
Source: Fungal Biol Biotechnol. 2014 Oct 14;1:4. doi: 10.1186/s40694-014-0004-9 (PMC5598268; doi:10.1186/s40694-014-0004-9)

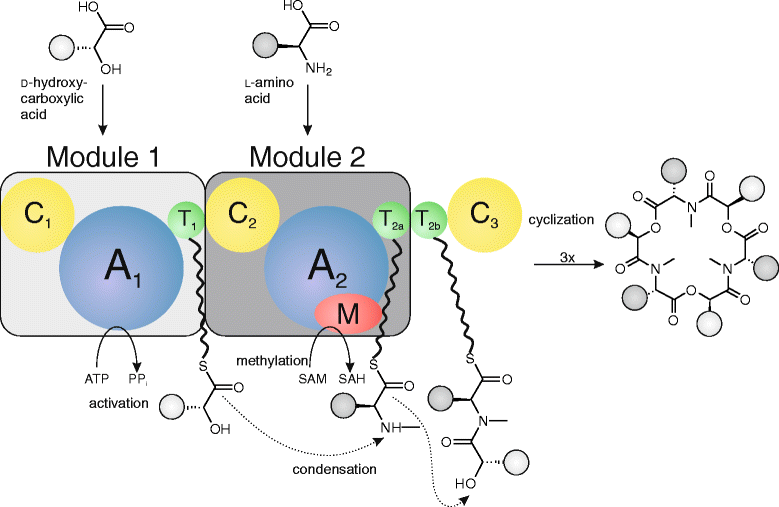

Supplement: Supplementary file 3 — Authors’ original file for figure 1 [file 40694_2014_4_MOESM3_ESM.gif]

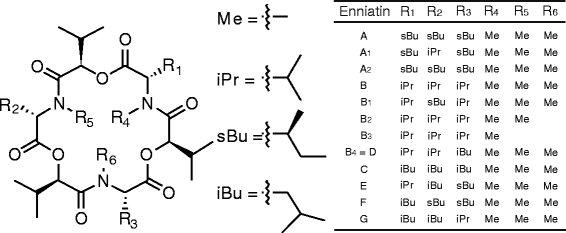

Supplement: Supplementary file 4 — Authors’ original file for figure 2 [file 40694_2014_4_MOESM4_ESM.gif]

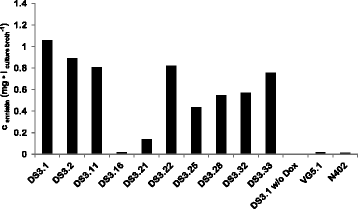

Supplement: Supplementary file 5 — Authors’ original file for figure 3 [file 40694_2014_4_MOESM5_ESM.gif]

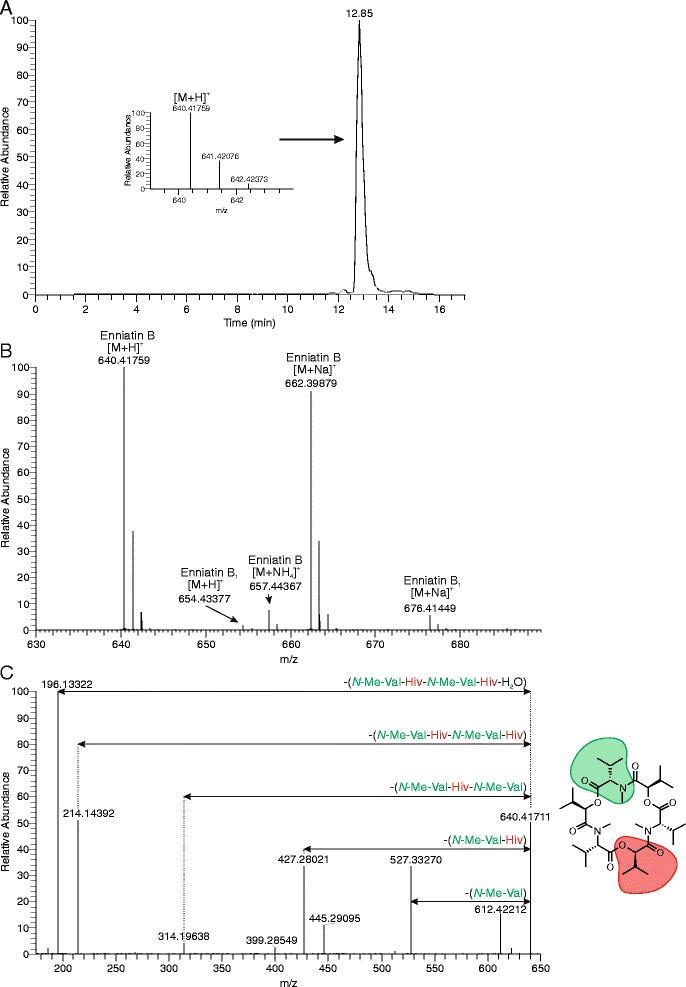

Supplement: Supplementary file 6 — Authors’ original file for figure 4 [file 40694_2014_4_MOESM6_ESM.gif]

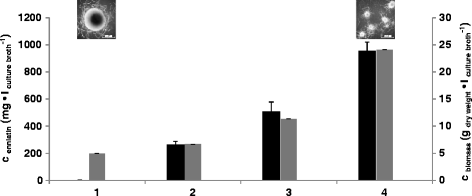

Supplement: Supplementary file 7 — Authors’ original file for figure 5 [file 40694_2014_4_MOESM7_ESM.gif]

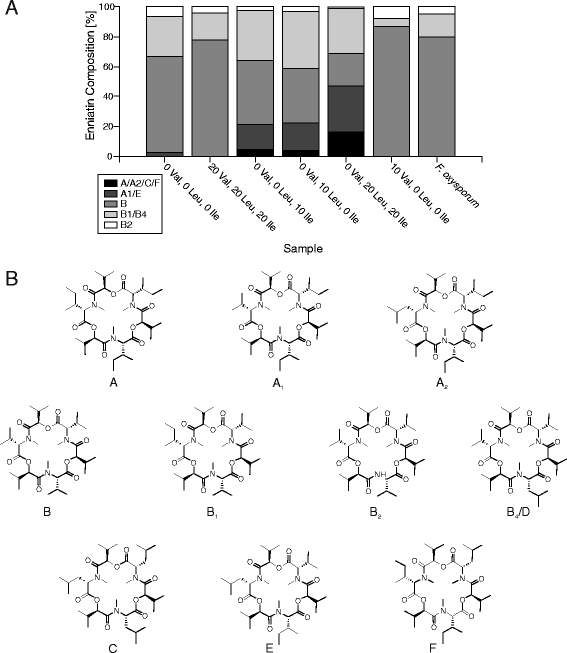

Supplement: Supplementary file 8 — Authors’ original file for figure 6 [file 40694_2014_4_MOESM8_ESM.gif]

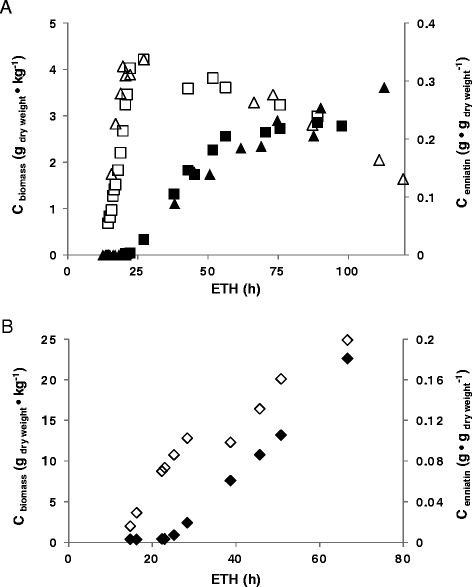

Supplement: Supplementary file 9 — Authors’ original file for figure 7 [file 40694_2014_4_MOESM9_ESM.gif]
